# Trade Subscriptions

**Published:** 1920-11-06

**Authors:** 


### Trade Subscriptions.

A CORRESPONDENT writes: "A special hospital in London lately enlisted the editor of a trade journal in support of an appeal. You noted the occasion at the time, and I have observed that the members of this trade in different parts of the country have their own hospital funds, and that in several districts the factories engaged in the same work, but not necessarily belonging to the same firm, unite to pay their hospital collections into a common fund, which is then distributed. In the main the impulse is a local one; and the recent appeal through the trade journal was interesting because it tried to influence an entire industry from the centre. Now that attempts are being made to organise regular contributions everywhere it is worth considerating whether these also might not be sought from the centre. In London such a plan would work in this way. The heads of those firms which have branches all over the city, and often outside it, would be approached to encourage a hospital collection in every branch of their business. By a little arrangement the hospitals in each neighbourhood where there was a branch could be encouraged to seek regular contributions at the same time, and the two impulses would probably lead to many new collections being formed throughout the country. Take, for instance, such retailers as Boots, cash chemists. Liptons, Lyons, the Home and Colonial and other national stores, W. H. Smith, the newsvendors, and all those businesses which meet us in every town, and in every quarter of the large towns, in England. Lord Hambleden, the head of W. H. Smith & Son, is a hospital chairman; Sir Jesse Boot, unless my memory fails, has given large sums to hospitals. What might not be expected if these gentlemen would encourage a hospital collection to be formed in every branch of their businesses? Their businesses employ in most cases a large staff, and their branches are so numerous that any example set by them would have a wide influence. The organisation of hospital collections has hitherto been mainly local. Why should not an impulse be given from the centre through the heads of firms like these which, in more than one instance, include men prominently identified with the voluntary system?"

Since the above words were written the press announces that 1,200 members of Messrs. W. H. Smith & Son have sent the proceeds of four weekly collections of 2d. per head to the funds of King's College Hospital. Could not the action of the head branch be followed by the sub-branches?

